# How Much PEEP Does High Flow Deliver via Tracheostomy? A Literature Review and Benchtop Experiment

**DOI:** 10.1155/2021/6036891

**Published:** 2021-01-13

**Authors:** Martin Thomas, Riddhi Joshi, Grant Cave

**Affiliations:** ^1^Department of Cardiology, Dubbo Base Hospital, Dubbo, NSW 2830, Australia; ^2^Department of Intensive Care, Tamworth Base Hospital, North Tamworth, NSW 2340, Australia; ^3^Department of Anaesthesia, Dubbo Base Hospital, Dubbo, NSW 2830, Australia; ^4^Dubbo Base Hospital, Dubbo, NSW 2830, Australia; ^5^Hawkes Bay District Health Board, Hastings, New Zealand

## Abstract

**Background:**

High flow tracheostomy (HFT) is a commonly used weaning and humidification strategy for tracheostomised patients, but little is known as to how much PEEP or mechanical benefit it offers. Patient anatomy and device characteristics differentiate it from high flow nasal cannula and the physiological effects observed.

**Objectives:**

(1) To review the available literature on the effects of HFT on airway pressure and indices of gas exchange. (2) To quantify PEEP generated by a HFT  circuit.

**Methods:**

A randomised benchtop experiment was conducted, with a size 8 uncuffed Portex tracheostomy connected to an Optiflow™ with Airvo 2™ humidifier system. The tracheostomy tube was partially immersed in water to give rise to a column of water within the inner surface of the tube. An air fluid interface was generated with flows of 40 L/min, 50 L/min, and 60 L/min. The amount of potential PEEP (pPEEP) generated was determined by the distance the water column was pushed downward by the flow delivered. *Findings*. Overall 40 L/min, 50 L/min, and 60 L/min provided pPEEP of approximately 0.3 cmH_2_O, 0.5 cmH_2_O, and 0.9 cmH_2_O, respectively. There was a statistically significant change in pPEEP with change in flows from 40–60 L/min with an average change in pPEEP of 0.25–0.35 cmH_2_O per 10 L/min flow (*p* value <0.01). *Interpretation*. HFT  can generate measurable and variable PEEP despite the open system used. The pPEEP generated with HFT is minimal despite statistically significant change with increasing flows. This pPEEP is unlikely to provide mechanical benefit in weaning patients off ventilatory support.

## 1. Introduction and Aim

High flow nasal cannula (HFNC) has become a mainstay of treatment in adult and paediatric ICU/HDU settings in the past decade [1]. HFNC has shown to improve respiratory mechanics and oxygenation by delivering up to 3–6 cm H_2_O of PEEP and increasing FRC by augmenting pulmonary airway pressures [2]. HFNC also causes flow dependant wash out of dead space CO_2_, with reductions in reinhaled CO_2_ [3]. HFNC has been observed to improve mucociliary clearance, reduce airway resistance, decrease work of breathing, and enhance neuro-ventilatory drive [4]. These physiological changes are subject to proper cannula fit, compliance of the patient to respire through the nasal passage and other factors such as patient anatomy [5].

High flow tracheostomy (HFT) is a common therapy utilised in delivering mechanical and physiological support to critical care patients. There is increasing incidence of tracheostomies being performed and managed in intensive care units over recent years [6]. Tracheostomy causes physiological change wherein the upper airway gets by passed. This diversion leads to elimination of PEEP offered by the upper airways, delivery of dry and cool air, and increase in thermal loss from the respiratory tract. The objective of HFT is to mitigate the physiological loss of an upper airway. Akin to HFNC, mechanical support with HFT targets the lost PEEP, previously offered by the upper airway. Similarly, use of the HFT along with a humidifier and temperature regulator ensures hydration of the respiratory tract along with maintenance of normothermia.

But unlike HFNC, HFT does not work with a closed-circuit system. HFT  is set up with a T-piece Mapleson system where one end remains open to facilitate exhalation. The question arises as to whether the same effects of HFNC are seen when humidified high flow is used in tracheostomised patients through a HFT. HFNC with an open mouth, leads to a 50% decrease in observed PEEP [7]. This replicates a HFT setting at a more distal respiratory anatomical setting. In theory, the physiological effects of HFNC cannot be expected to be matched by HFT. PEEP is lost both with bypass of the upper airway and a system open to the atmosphere [8]. The effect of dead space washout would be expected to be lower, with a reduced anatomical dead space in tracheostomised patients. While much research has been done on HFNC in clinical settings, relatively few studies have explored the effectiveness of HFT.

This study has the following 2 aims:

- To review available literature on the effects of HFT on airway pressure and indices of gas exchange.
- To measure potential PEEP (pPEEP) generated by a HFT circuit. We additionally tested whether pPEEP differed with increasing gas flows against the null hypothesis that pPEEP generated would not change due to mechanical limitations of the high flow system.

## 2. Literature Review

We undertook a literature search of PubMed and the Cochrane Library databases using search terms “High Flow” AND “Tracheostomy” in accordance with the PRISMA statement [9]. Studies were included if they included high flow in a tracheostomy setting and measured airway pressures and/or indices of gas exchange. Studies were excluded if high flow was applied via nasal prongs or if neither airway pressures nor gas exchange indices were reported. 109 search results were obtained from PubMed and 31 were obtained from the Cochrane Library.

**Figure 1:**
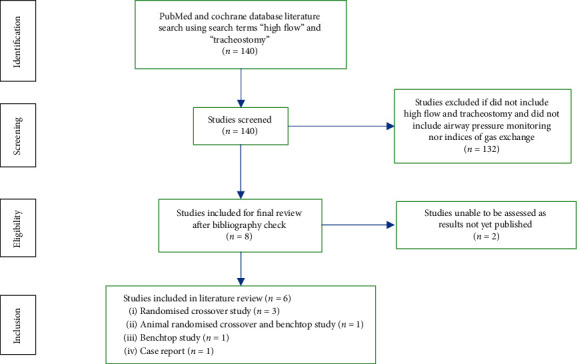
PRISMA diagram to outline selection of cases for literature review.

After reviewing the articles, the bibliography, and exclusion of unrelated studies, 6 were chosen to be reviewed as outlined in Figure 1. Of the 6 studies, 3 clinical studies explored HFT and its mechanical benefits on the respiratory system, 1 animal study explored a modification of the HFT in pigs, 1 benchtop experiment demonstrated effects of flow and resistance in different placement of tracheostomy tubes, and 1 case series evaluated effectiveness of HFT in weaning patients with restrictive lung disease from prolonged mechanical ventilation.

Few studies have explored how much airway pressure HFT delivers and the difference in airway pressure with various gas flows. Corley et al. compared 20 tracheostomised patients in a randomised crossover study with HFT at 50 L/min and T piece at 15 L/min (15-minute treatment period each). They demonstrated statistically significant differences in SpO_2_/FiO_2_ and mean airway pressure after 15 minutes of HFT (SpO_2_/FiO_2_ ratio of 244.4 in HFT vs. SpO_2_/FiO_2_ ratio of 210.3 in T piece and 0.7 cm H_2_O difference in mean airway pressures between T piece and HFT *p* value 0.01 for both variables) [10].

In a single centre randomised crossover study, Natalini et al. compared standard oxygen therapy (O_2_ flow at 8 L/min) and HFT with flows of 10 L/min, 30 L/min, and 50 L/min (30-minute treatment period each). Statistically significant differences in oxygenation, respiratory rate, peak, and mean expiratory pressures were observed with 50 L flow but not with reduced flows. The study also compared the difference between HFT and HFNC in a subgroup of 5 patients who underwent tracheostomy decannulation and determined that in comparison to HFNC, the changes observed in HFT were significantly less. At 50 L/min flow, the median peak expiratory pressure with HFNC was 5.1 cmH_2_O vs. 1.8 cmH_2_O with HFT and the mean expiratory pressure was 3.9 cmH_2_O with HFNC vs. 1.5 cmH_2_O with HFT [11].

In a single centre unblinded crossover study in 14 tracheostomised patients, Stripoli et al. explored the association of HFT (50 L/min flow) with neuro-ventilatory drive, work of breathing, and gas exchange. EAdi (electrical activity of diaphragm), Pmusc (pressure generated by inspiratory muscles), respiratory rate, and ABG analysis were undertaken. Patients were treated with two 1-hour periods of high flow alternated with an hour of conventional oxygen therapy (T-piece at 10 L/min). Their study demonstrated that there was no difference observed between conventional and HFT for any variable, implying that HFT may not help in patients at high risk of weaning failure [12].

Chen et al. evaluated the effects of HFT at 10 L/min, 40 L/min, and 40 L/min with a modified interface (a 5 cm H_2_O/L/Sec resistor on the expiratory port) in 6 tracheostomised pigs. Respiratory mechanics and oxygenation indices were compared for the three gas delivery methods in a randomised crossover fashion with 20 minutes for each mode. All modes were instituted before and after inducing mild lung injury with surfactant depletion. Only the modified HFT setting generated statistically significantly differences in end expiratory lung volume, inspiratory and expiratory pressures in both the normal and injured lung models. The PaO_2_/FiO_2_ ratio was also significantly higher in the modified HFT group in the injured lung model [13].

In a benchtop experiment, Moorhouse et al. explored the changes in tracheal airway pressures with properly and improperly placed tracheostomy tubes using HFT gas delivery. Mean airway pressures recorded for appropriately placed cuffed tracheostomy tubes during the study were 0.15 cm H_2_O, 0.47 cm H_2_O, and 0.94 cm H_2_O for 10 L/min, 20 L/min, and 30 L/min flows, respectively. Airway resistance and pressures increased with improper placement of tracheostomy tubes [14].

Mitaka et al. described 2 cases where HFT was used to apparent effect in weaning patients with restrictive lung pathology from ventilatory support [15]. In the first case, a 78-year-old man with postoperative pneumococcal pneumonia, who subsequently developed restrictive lung dysfunction (*C*_static_ 14–15 mL/cmH_2_O, TV 230–240 ml) was successfully weaned off ventilator support over 16 days with HFT at 40 L/min. The second case was a 69-year-old male who developed restrictive lung dysfunction (*C*_static_ 16–17 mL/cmH_2_O, TV 210–220 ml) after aspiration pneumonia. He was successfully weaned off ventilatory support after 12 days of HFT therapy. This patient exhibited reversible hypercapnoea when HFT was substituted for T-piece supplemented with 3 L/min oxygen.

With the growing number of tracheostomies in mechanically ventilated patients, HFT will be increasingly considered as a weaning strategy [16]. To the best of our knowledge, there is no published data that has quantified PEEP delivered by HFT in tracheostomised patients. The scarcity of literature in this area leads to hesitation in clinicians in using this mode of ventilation as a weaning strategy. More research is needed to assess the utility and validate the use of HFT in clinical practice. Two upcoming trials may answer some of these questions. A trial from Kobe University, Japan, will explore the effect of HFT to airway pressure in postoperative tracheostomised patients [17]. Another study being undertaken in Siriraj Hospital, Thailand, aims to decipher the physiologic effects of HFT in patients requiring mechanical ventilation [18]. Description of studies included in the review is listed in Table 1.

This review demonstrates a paucity of evidence in the published literature on the mechanical benefits offered by HFT. In addition, although most of these studies have demonstrated statistically significant increases in airway pressures with increase in flows, the pressure augmentation with HFT appears to be low. This may be explained by the open gas delivery system employed in HFT, where the gas mixture flows to the tracheostomy tube, but as it encounters an airway pressure greater than the blowoff pressure of the circuit, it redirects to vent to the less resistive exterior. Augmentation of airway pressure with HFT at a time of the respiratory phase where there is no gas flow, as is the case with end expiratory pressure, cannot be greater than the blowoff pressure of the open gas delivery system. We determined to measure this pressure, which we term potential PEEP (pPEEP) in a benchtop experiment.

### 2.1. Benchtop Experiment

We conducted an in vitro laboratory experiment to quantify and study the PEEP generated by HFT. From patient to instruments, the various factors that influence generation of PEEP in HFT are listed in Table 2. To evaluate the independent contribution of HFT in generation of PEEP, we ascertained that an in vitro laboratory experiment was most appropriate. While PEEP generation involves a complex interplay of airway mechanics and instrument factors, large airway pressures at a point in the respiratory phase of no airflow *in vivo* would not be augmented by more than pPEEP as measured in the experiment. The same was observed by Bailes et al. in a study comparing nasal high flow vs. bubble CPAP [19]. Keeping this in mind, we were able to test the maximum potential PEEP that could be generated by different flows in a HFT setting.

## 3. Methodology

**Figure 2:**
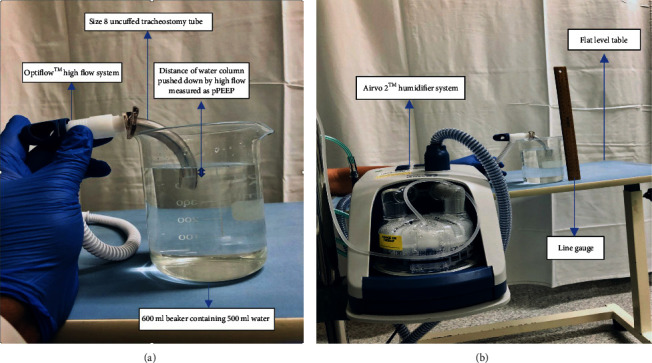
(a) Image of benchtop experiment setup. (b) Airflow™ humidifier with Optiflow™ high flow circuit.

The experiment was undertaken in October 2018 at the Tamworth Base Hospital Intensive Care Unit. Ethics approval was not sought given no patient involvement. The apparatus involved in the benchtop experiment included the following, depicted in Figures 2(a) and 2(b):

1. A size 8 uncuffed Portex tracheostomy tube
2. Fischer and Paykell Optiflow™ high flow circuit
3. Airvo 2™ humidifier system with oxygen connected through a wall outlet
4. 600 ml beaker containing 500 ml water, stabilised on a flat level table
5. A line gauge to measure the column of water pushed down by respective flows

The gas flow rate was randomised using a random number generator by an operator independent of flow measurement. A size 8 Portex tracheostomy tube was attached to an Optiflow™ with Airvo 2™ humidifier system with oxygen connected through a wall outlet. The tracheostomy tube was partially immersed in the beaker to 3 centimetres by hand to give rise to a column of water within the inner surface of the tube. The apparatus was stabilised by a sturdy flat level table. In addition, the tracheostomy tube was partly stabilised using the phalanges of the tracheostomy tube over the rim of the beaker. As flows were initiated, an air fluid interface was generated within the inner surface of the tracheostomy tube. The amount of pressure generated was determined by the distance the water column was pushed downward by the flow delivered, analogous to the prescription of PEEP with bubble CPAP [19].

The observed depth of the air column in water was recorded as pPEEP (potential PEEP). Measurements were recorded 1 minute after the initiation or change in flow; this was done to allow the system to equilibrate to the change in flow and to avoid error. The pPEEP generated was measured by two independent observers with the help of a line gauge for accuracy. The observers were blinded to the amount of flow during each reading, and the recorder was blinded to the measurement of pPEEP. The measurements were verbally relayed to the recorder by the two observers, and an average of the two readings was documented for accuracy.

### 3.1. Statistical Analysis

The Shapiro–Wilk test was used to assess for normality in the distribution of data. Parametric testing with ANOVA/*t*-test and nonparametric testing using the Kruskal–Wallis H/Mann–Whitney *U* test was used as appropriate. 5 preliminary measurements at another centre using a different investigator gave an estimate of the standard deviation of the PEEP measurement using this technique of 0.5 mm. The benchtop study was powered at >90% to detect a PEEP difference of 0.1 cmH_2_O (the minimum measurable difference) between flow rates at an alpha of 0.05 with 20 recordings in each group [20]. A set of 60 readings of pPEEP were recorded, with 20 readings each at flow rates of 40 L, 50 L, or 60 L. Statistical analysis was done using IBM SPSS Statistics for Macintosh, version 23.0 (IBM Corp., Armonk, NY, USA).

## 4. Results

A total of 60 observations, 20 with each flow, were recorded during the entire experiment. pPEEP was measured in cmH_2_O and was determined by the distance the column of water was pushed down by the corresponding flow. Descriptive statistics for different flow rates are listed in Table 3. Overall, 40 L/min, 50 L/min, and 60 L/min provided pPEEP of approximately 0.3 cmH_2_O, 0.5 cmH_2_O, and 0.9 cmH_2_O, respectively.

**Figure 3:**
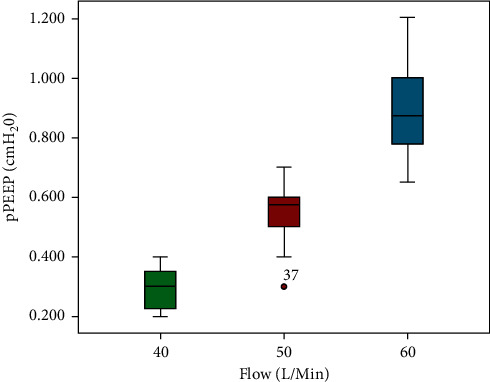
Box plots of pPEEP in cmH_2_O with 40 L/min, 50 L/min, and 60 L/min flows.

**Figure 4:**
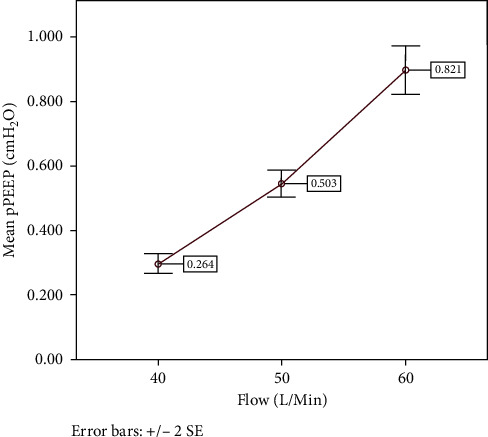
Mean and SE of pPEEP in cmH_2_O with 40 L/min, 50 L/min, and 60 L/min flows.

Data were nonnormally distributed (*p* = 0.019, Shapiro–Wilks). Nonparametric analysis with Kruskal–Wallis H test revealed statistically significant differences in pPEEP between the three flow groups. Similarly, post hoc analysis using Mann–Whitney U test to check significance when two groups compared with each other revealed statistical significant difference in pPEEP (*p* value <0.01) with all combinations of flow. Figures 3 and 4 are box plots and standard error (SE) charts of the pPEEP observed with the different flow rates.

## 5. Discussion

While the effects of HFT on patient-centred outcomes remain undefined, the sum of the available literature and our benchtop experiment suggest that effects on airway pressures in HFT may not provide a mechanism for clinically meaningful outcomes. Our experiment showed that HFT generates approximately 0.3, 0.5, and 0.9 cmH_2_O of pPEEP with flows of 40 L/min, 50 L/min, and 60 L/min, respectively. The magnitude of these measures corresponds reasonably with previous studies of airway pressure in previous studies [13, 15].

Our study demonstrated that there was a statistically significant change in pPEEP with change in flows from 40–60 L/min with an average change in pPEEP per 10 L/min flow of 0.25–0.35 cmH_2_O. The null hypothesis that increasing flows would not lead to changes in pPEEP with HFT was rejected, stating that HFT can generate measurable and variable PEEP despite the open system used. While these findings were statistically significant, changes in airway pressure of this magnitude do not seem likely to be clinically meaningful. The capacity for this amount of PEEP to translate into increased airway pressures, increased functional residual capacity (FRC), increased arteriolar oxygen tension (PaO_2_) and displacement of extravascular lung water is questionable [19].

There are studies of clinically important endpoints for HFT in the literature. High flow rates (>50 L/min) may improve oxygenation, as suggested by increases in SpO_2_/FiO_2_ and PaO_2_/FiO_2_ in studies by Corley et al. and Natalini et al., respectively [10, 11]. Natalini et al. reported improved work of breathing in the form of reduced respiratory rate with flows of 50 L/min vs. standard oxygen therapy up to 8 L/min (24 [21–29] breaths/min vs. 26 [22–33] breaths/min, *p*=0.02) [11]. This is likely related to reduced entrainment of atmospheric air with increased flows. Entrainment of air depends on the patient's inspiratory flow rate and the delivered flow. In acute respiratory failure, inspiratory flow rate requirements can go up to 120 L/min. Lower flow rates in these scenarios lead to entrainment of air, leading to less accurate PEEP and FiO_2_ delivery.

Work of breathing is a complex interplay of elastic and resistive forces and requires neural integration. Respiratory rate is only one of the components of work of breathing. Stripoli et al., who had more holistic variables in determining the effect of HFT on work of breathing, found that the EAdi (electrical activity of diaphragm), inspiratory PTP (pressure time product), respiratory rate, and blood gas remained unchanged with HFT as compared to standard oxygen therapy [12]. Currently, there is insufficient evidence to claim HFT reduces the work of breathing.

Natalini et al. hypothesized that the already reduced dead space and more efficient CO_2_ clearance in tracheostomised patients limits the benefits from HFT compared to HFNC. The hypothesis is consistent with the findings of all the three studies (Corley et al., Natalini et al., and Stripoli et al.) which reported no significant effect in CO_2_ clearance in HFT patients, against reported increased CO_2_ clearance with HFNC in COPD patients [3, 10–12]. Evidence for improved CO_2_ clearance with HFT is limited to the Mitaka case report. The authors suggest the presence of restrictive lung disease may have played a role [15]. Whether HFT offers an advantage for restrictive lung pathology where any dead space washout is likely to be proportionally greater may be explored in future work.

The strengths of the benchtop study include an important clinical question and sound physiological plausibility. The study experiment was simple, easy to setup and conduct, and highly reproducible. Blinding of observers to flow and blinding of the recorder during the process of PEEP measurement increase internal validity. pPEEP was measured in cmH_2_O which was an easy to measure output, unlike other experiments that measured complex variables. Although the study lacked patient exposure, this helped avoid other confounders encountered in clinical PEEP measurement. The extrinsic PEEP measured in a clinical scenario would be lower and cannot exceed the measurements observed in this experiment, which can be attributed to the resistance encountered during spontaneous breathing and to matching of flows to meet inspiratory demand [19].

The study did not include direct measurement of PEEP in patients exposed to HFT. While we believe the rationale that augmentation of airway pressure cannot exceed the “blowoff” pressure of the delivery system, the *in vitro* nature of our work may limit the extrapolation of the study to a clinical setting. Study limitations include measurement errors due to parallax shift, refractive errors of light passing through different media and operator/observer error. Mitigation of measurement error by usage of advanced manometric monitors is a limitation of the study. Observer bias was a potential study limitation, as measurements were verbalised to the operator without concealment. Another limitation could be that surface tension changes and airway resistance changes with different sized and types of tracheostomy tubes which were not taken into account [21]. The radius of curvature of the tracheostomy tube would have led us to underestimate the PEEP generated, as the column of fluid pushed down was measured using vertical line gauges. A further limitation of our study was that the apparatus was not completely stabilised during measurements of pPEEP.

## 6. Conclusion

Despite the open system attributed to HFT, the experiment demonstrates variable and statistically significant increase in pPEEP with increments of flow. However, the pPEEP generated even with highest flow is low and appears unlikely to provide mechanical assistance in a patient being weaned off ventilatory support. Further *in vivo* research is required to ascertain the clinical impact of pressure augmentation with HFT and to determine whether HFT translates to positive patient-centred outcomes.

## Figures and Tables

**Table 1:** Description of studies included in review.

Author (year)	Study type	No. of patients/subjects	High flows administered	Variables measured	Results	Statistically significant variables
Corley et al. (2017)	Randomised crossover study (HFT vs TPiece)	20	50 L/min	EELV, TV, paw (mean), SpO_2_/FiO_2_, EtCO_2_, RR, HR, dyspnoea score	Mean airway pressures 0.7 cmH_2_O; mean difference between HFT and T Piece	SpO_2_/FiO_2_, Mean Airway Pressure

Natalini et al. (2019)	Single centre randomised crossover study (HFT vs standard oxygen)	26	10 L/min, 30 L/min, 50 L/min	ABG/ RR / peak and mean expiratory pressure	Peak expiratory pressures 1.8 cmH_2_O (1.4–2.2 cmH_2_O) with 50 L/min vs. 1.3 cmH_2_O (0.9–2 cmH_2_O) with standard oxygen, p=0.001 Mean expiratory pressure 1.2 cmH_2_O (1–1.5 cmH_2_O) with 50 L/min vs. 0.8 cmH_2_O (0.5–1.3 cmH_2_O) with standard oxygen, p<0.001	PaO_2_/FiO_2_, RR, peak and mean expiratory pressures

Stripoli et al. (2019)	Single centre unblinded crossover study (HF1/oxygen/HF2)	14	50 L/min	ABG/RR/EAdi/Pmusc/ work of breathing	No airway pressure was monitored	None

Chen et al. (2019)	Animal randomised crossover study/bench experiment (TPiece/HFT/modified HFT)	6	40 L/min for animal study, 10 L/min–60 L/min for bench study	ABG/RR/paw/PEEP/PTP/resistance	Animal study bench experiment 1± 0.3 cmH_2_O (mean paw) 40 L/min 1.2 ± 0.3 cmH_2_O (mean paw) 50 L/min 1.5 ± 0.3 cmH_2_O (mean paw) 60L /min	Paw, resistance, EELV, PaO_2_/FiO_2_

Moorhouse et al. (2015)	Benchtop experiment	468 Recordings	5 L/min to 30 L/min in increments of 5 L/min	Airway pressure and resistance to flow	0.15 cmH_2_O for 10 L/min (paw) 0.47 cmH_2_O for 20 L/min (paw) 0.94 cmH_2_O for 30 L/min (paw)	Resistance and pressure

Mitaka et al. (2018)	Case report	2	40 L/min	Airway pressure	Case 2 0.21–0.3 cmH_2_O for 10 L/min (paw) 0.21–0.56 cmH_2_O for 20 L/min (paw) 0.54–0.91 cmH_2_O for 30 L/min (paw) 0.76–2.01 cmH_2_O for 40 L/min (paw) 1.17–2.01 cmH_2_O for 50 L/min (paw) 1.76–2.01 cmH_2_O for 60 L/min (paw)	NA

Abbreviations: EELV: end expiratory lung volume; TV: tidal volume; Paw: airway pressure; SpO_2_: oxygen saturation; FiO_2_: fraction of inspired oxygen; EtCO_2_: end tidal CO_2_; RR: respiratory rate; HR: heart rate; HFT: high flow tracheostomy; cmH_2_O: centimetres of water; ABG: arterial blood gas; Pmusc: pressure generated by inspiratory muscles; EAdi: electrical activity of the diaphragm; PTP: pressure time product; PEEP: positive end expiratory pressure; PaO_2_: partial pressure of oxygen.

**Table 2:** Factors affecting PEEP in HFT.

Patient factors	(i) Age/gender (ii) Lung volumes/capacity and respiratory rate (iii) Respiratory tract dimension (iv) Respiratory tract resistance (v) Respiratory tract compliance (vi) Anatomical and pathological variations of respiratory tract (vii) Percentage of respiratory lumen occupied by tracheostomy tube determines amount of leak and patient comfort

Instrument factors	(i) Flow and density of air oxygen mixture (ii) Length, diameter, and type of tracheostomy tube used (iii) Angle between axis of connector and delivery tube (60° for Fischer and Paykell Optiflow™) determines turbulence of flow (iv) Pressure at the exhalation port, as this determines the blowoff pressure. Normally, this is atmospheric pressure

**Table 3:** Descriptive statistics comparing flows.

Flow	40 L/min	50 L/min	60 L/min
No. of observations	20	20	20
Mean pPEEP (cmH_2_O)	0.295	0.545	0.898
Median (IQR) (cmH_2_O)	0.3 (0.213–0.35)	0.58 (0.5–0.6)	0.88 (0.763–1.0)
Mode (cmH_2_O)	0.350	0.6	0.9
Standard deviation (cmH_2_O)	0.069	0.094	0.172
Standard error of mean (cmH_2_O)	0.015	0.021	0.038

## Data Availability

Data and analysis to support the findings can be obtained from the corresponding author Dr. Martin Thomas (Ph: +61432645290 and e-mail: martinjosethomas@gmail.com).
